# Activation of endothelial cells by extracellular vesicles derived from *Mycobacterium tuberculosis* infected macrophages or mice

**DOI:** 10.1371/journal.pone.0198337

**Published:** 2018-05-31

**Authors:** Li Li, Yong Cheng, Scott Emrich, Jeffrey Schorey

**Affiliations:** 1 Department of Biological Sciences, Eck Institute for Global Health, Center for Rare and Neglected Diseases, University of Notre Dame, Notre Dame, IN, United States of America; 2 Department of Electrical Engineering and Computer Science, University of Tennessee, Knoxville, TN, United States of America; Rutgers Biomedical and Health Sciences, UNITED STATES

## Abstract

Endothelial cells play an essential role in regulating an immune response through promoting leukocyte adhesion and cell migration and production of cytokines such as TNFα. Regulation of endothelial cell immune function is tightly regulated and recent studies suggest that extracellular vesicles (EVs) are prominently involved in this process. However, the importance of EVs in regulating endothelial activation in the context of a bacterial infection is poorly understood. To begin addressing this knowledge gap we characterized the endothelial cell response to EVs released from *Mycobacterium tuberculosis* (*Mtb*) infected macrophages. Our result showed increased macrophage migration through the monolayer when endothelial cells were pretreated with EVs isolated from *Mtb*-infected macrophages. Transcriptome analysis showed a significant upregulation of genes involved in cell adhesion and the inflammatory process in endothelial cells treated with EVs. These results were validated by quantitative PCR and flow cytometry. Pathway analysis of these differentially expressed genes indicated that several immune response-related pathways were up-regulated. Endothelial cells were also treated with EVs isolated from the serum of *Mtb*-infected mice. Interestingly, EVs isolated 14 days but not 7 or 21 days post-infection showed a similar ability to induce endothelial cell activation suggesting a change in EV function during the course of an *Mtb* infection. Immunofluorescence microscopy result indicated that NF-κB and the Type 1 interferon pathways were involved in endothelial activation by EVs. In summary, our data suggest that EVs can activate endothelial cells and thus may play an important role in modulating host immune responses during an *Mtb* infection.

## Introduction

Extracellular vesicles (EVs) are important mediators of intercellular communication and are known to carry all the different macromolecules: proteins, carbohydrates, lipids and nucleic acids. Their complex composition allows for engagement of multiple receptors and transfer of numerous cellular components resulting in a marked change in the recipient cell. EVs consist of three major forms: apoptotic bodies, microvesicles, and exosomes. Microvesicles bud from the plasma membrane while exosomes are released from cells upon fusion of a multivesicular body with the plasma membrane and release of the intraluminal vesicles. The composition and function of the different EVs varies and depends on the cell of origin and the physiological state at the time of EV release. Recent studies have focused on the role of EVs in the context of disease pathogenesis and their production and function has been linked to a number of diseases including cancer, cardiovascular and infectious diseases [1, 2]. A significant effort has also focused on EVs as potential biomarkers for various diseases [3–7].

EVs released from mycobacteria-infected macrophages are known to contain mycobacterial components including PAMPs (Pathogen-associated Molecular Patterns) and can stimulate the production of pro-inflammatory molecules such as TNF-α and RANTES in recipient macrophages [8]. Some of these PAMPs may also be associated with bacterial membrane vesicles released from *M*. *tuberculosis* during an infection of its host macrophage (9). EVs isolated from serum of mice infected with *Mycobacterium tuberculosis* (*Mtb*) can also active macrophages *ex-vivo* [9]. However, the effect of these EVs on other potential recipient cells has not been assessed. One potential target is endothelial cell, as EVs are present in circulation. Moreover, during an *Mtb* and *M*. *bovis* BCG mouse infection, the concentration of circulating EVs increases leading to elevated exposure of endothelial cells to these vesicles [10, 11].

Endothelial cells are known to play an important role in responding to a microbial infection [12]. During gram-negative bacterial infections circulating LPS result in activation of the nuclear factor (NF)-κB transcription factor in endothelial cell leading to upregulation of leukocyte adhesion molecules and increased cell permeability [13]. This upregulation leads to enhanced immune cell adhesion and cell migration [14,15]. Endothelial cells are both the producers and recipients of EVs and the presence of EVs in circulation can have a significant effect on vascular function including effects on angiogenesis and vascular repair [16]. EVs activity has also been closely linked to atherosclerotic plaque formation through, for example, promoting monocyte adhesion to endothelial cells [1]. However, the role of EVs in regulating endothelial cell function during an infection has remained relatively undefined and the studies that have been published focus on viral pathogens [17]. Hepatitis C infection, for example, has been linked to type I and type III IFN production by infected liver endothelial cells and EVs from IFN-β exposed liver sinusoidal endothelial cells can inhibit viral replication [18]. In contrast, how endothelial cell function is affected by EVs generated during a bacterial infection has not been studied but are warranted as endothelial cells are important in facilitating an immune response against bacterial pathogens such as *Mtb*. Our present work indicates that EVs released from *Mtb*-infected macrophages or isolated from the serum of infected mice can activate endothelial cells leading to increased expression of cell adhesion molecules, chemokines and chemokine receptors as well as promote macrophage migration.

## Materials and methods

### Ethics statement

The University of Notre Dame is accredited through the Animal Welfare Assurance (#A3093-01). All animals and procedures in this study were approved by the University of Notre Dame Institutional Animal Care and Use Committee under the protocol entitled Mycobacterial-Host Cell Interaction and Disease Pathogenesis. The mice in this study were observed daily by the veterinary staff and any mice that show abnormal body posture, lack of grooming, etc. as defined in the University of Notre Dame’s Freimann Life Science Center Human Endpoint SOP were euthanized using a CO^2^ euthanex chamber. Mice were housed in cages containing appropriate bedding and environmental enrichment (e.g. plastic tubing) and were given food and water *ad libitum*.

### Endothelial and macrophage cell culture and H37Rv growth culture

The mouse endothelial cell line SVEC4-10 (ATCC) was cultured in DMEM (Dulbecco's Modified Eagle Medium) media with 10% fetal bovine serum (FBS) as recommended. The mouse macrophage cell line RAW264.7 (ATCC) was maintained in DMEM media supplemented with 10% FBS. Bone marrow-derived macrophages (BMMs) were isolated from 6- to 8-wk old C57BL/6 mice. Bone marrow was isolated, and fibroblasts and mature macrophages were removed by selective adhesion. Bone marrow was cultured at 37°C in the presence of 5% CO2 in DMEM supplemented with 10% fetal bovine serum (GIBCO BRL), 20 mM HEPES (Fisher Scientific), 100 U of penicillin per ml, 100 μg of streptomycin (BioWhittaker) per ml, and 15% L-cell supernatant as a source of macrophage colony-stimulating factor. After 4 days in culture, macrophages were supplied with fresh media, and mature macrophages were used on day 7 of culture. Bacteria colonies recovered from the spleen of C57BL/6 mice two weeks post-infection with *Mtb* H37Rv were grown in Middlebrook 7H9 broth supplemented with oleic albumin dextrose catalase (OADC) until mid-logarithmic phase (OD600 ~1.0) and frozen in growth media with 15% glycerol. Prior to use, the bacterial stocks were thawed and the mycobacteria were de-clumped by passage through a 27-gauge needle 10x.

### Isolation of EVs from cell culture supernatants

Confluent monolayers of RAW264.7 (~ 1x10^7^ cells) were seeded overnight in Ti175 flasks and infected with *Mtb* or were left un-infected. Prior to infection, the bacteria were complement opsonized in DMEM media supplemented with 10% normal horse serum for 2hrs. A MOI of 5:1 was used to obtain about ~80% of the RAW264.7 cells infected as described [10, 19]. RAW264.7 cells were infected for 4 hrs, washed 3x with PBS to remove extracellular free bacteria and then cultured in DMEM media with 10% EV-free FBS (overnight spin to deplete EVs in serum) for 72hrs. EVs were isolated from the culture supernatants of infected and uninfected RAW 264.7 cells by centrifugation at 3,000×g for 10 mins to remove cell debris followed by filtration twice through 0.22μm filter (Thermo Fisher Scientific). The supernatant was further centrifuged at 100,000×g for 1hr at 4°C. The pellets were washed once and resuspended in PBS.

### Permeability assay

SVEC4-10 cells were seeded on the top chamber of the Transwell plate (Costar, Corning, NY; 0.4μm pore size polycarbonate membrane filter) coated with 6–10 μg/cm^2^ of type 1 collagen (Sigma) and cultured for 6 days. The cell numbers needed to obtain an intact monolayer was determined by measuring the cell concentration that could block >95% of the 70kD Rhodamine-labeled dextran (EX: 520nm, EM: 590nm) diffusing to bottom chamber of transwell plate. Detection of Rhodamine-B labeled dextran was performed using a SpectraMax M5 microplate reader (Molecular Devices). Cells were left untreated or stimulated with 40 μg/ml of EVs derived from uninfected or *Mtb*-infected RAW264.7 cells (equal to ~6 x 10^11^ EVs). 4kD FITC-labeled dextran or 70kD Rhodamine-labeled dextran was added to top chamber. Culture medium was removed from bottom chamber at indicated times and concentration of dye in the media measured using the SpectraMax M5 microplate reader.

### RNA isolation, cDNA synthesis and qPCR validation

Endothelial cells were left untreated or treated for 4hrs with 40μg/mL (quantified by BCA assay) of EVs derived from uninfected or *Mtb*-infected RAW264.7 cells. Total RNA was isolated using QIAshredder column (Qiagen) and RNeasy mini kit (Qiagen). cDNA was synthesized (SuperScript™ III First-Strand Synthesis System from Thermo Fisher Scientific) and qPCR was performed using PerfeCTa SYBR Green SuperMix (Quanta Biosciences) and samples were run on AB7500 Fast Cycler (Thermo Fisher Scientific) following the manufacturer’s instructions. Relative RNA expression was normalized to endogenous reference gene, *gapdh* and was quantified using the comparative Ct method with the formula 2^-ΔΔCt^. Primer sequences are shown in S1 Table.

### Mouse infections, EV isolation and CFU determination

C57BL/6 mice obtained from the Freimann Life Science breeding colony were infected retro-orbitally with 2x10^6^ *Mtb* or injected with an equal volume of PBS. A total of 32 mice were used with four mice per time point and one control uninfected group. Mice were used for the isolation of blood by heart puncture and the spleens removed for quantifying bacterial load. Mice were anesthetized by CO_2_ prior to the heart puncture. EVs were isolated from mouse serum by filtration through 0.45μm filter followed by 0.22μm filter, and the serum was further centrifuged at 100,000×g for 1hr at 4°C. Spleens were homogenized and passed through a 70μm cell strainer to remove tissue. Spleen homogenates were processed as described (9) and plated on 7H10 agar (Dot Scientific) supplemented with 10% OADC and Tween 80 (0.05% v/v). CFUs were determined after 4 wks.

### NanoSight

The EVs were quantified by NanoSight LM10 (Malvern) using the 635 nm red laser and NTA 3.2 analytical software as previously described [10].

### *In vitro* Transwell cell migration assay

SVEC4-10 cells were seeded as described above to form intact monolayers. Cells were left untreated or stimulated for 3 hrs with EVs isolated from uninfected, *Mtb*-infected RAW264.7 cells (40μg/mL, measured by BCA assay) or from C57BL/6 mouse serum (2.5x10^12^, determined by vesicle number using NanoSight to rule out a potential inaccurate quantification caused by serum enriched proteins). BMMs labeled with 2.5μM Carboxyfluorescein succinimidyl ester (CFSE) (Molecular Probes) were added in the top chamber of the transwell (Costar) and allowed to adhere and migrate for 4hrs. The filters were rinsed gently with PBS to remove unattached macrophages and placed on a slide mounted with a coverslip. Fluorescent macrophages that migrated to the bottom surface of the Transwell filter were counted in 7 randomly selected fields, using a Zeiss Observer fluorescent microscope.

### Flow cytometry

SVEC4-10 cells were seeded in collagen-coated 6 or 24-well plates (3 × 10^5^ or 0.5 × 10^5^ cells per well respectively) and allowed to culture for 3 days to form monolayers. The cells were left untreated or stimulated with EVs from uninfected or *Mtb*-infected RAW264.7 cells and C57BL/6 mouse serum for 16hrs. For cell surface staining, the cells were washed in FACS buffer and blocked with 10% normal mouse serum and stained with FITC-labeled rat anti-mouse VCAM1 (Biolegend) or FITC labeled anti-rat IgG2a antibody (Biolegend) as isotype control (1/200) or FITC conjugated anti-mouse TLR2 antibody (Biolegend) or FITC conjugated anti-mouse IgG1 antibody as isotype control (1/200) (BioLegend). For CCL2 antibody staining cells were treated with 2μM monensin solution (BioLegend) for 2hrs after EV treatment to block protein secretion from the cell. The cells were washed with PBS and detached, using 0.25% Trypsin-EDTA (Gibco), followed by a PBS wash. For intracellular staining, cells were fixed and permeabilized with permeabilization wash buffer (Biolegend) followed by blocked in 10% normal mouse serum and further stained with 1/40 diluted PE-conjugated Armenian Hamster anti-mouse CCL2 (Biolegend) or 1/40 diluted PE-labeled Armenian Hamster IgG as isotype control (BioLegend). The protein expression was analyzed using a Beckman Coulter flow cytometer (FC 500 Series), and the data analyzed using CXP Analysis software.

### Immunofluorescence confocal microscopy for NF-κB

SVEC4-10 cells (8 × 10^3^ cells per well) were seeded in collagen-coated cover slips in 24-well plates and cultured for three days. Cells were treated with EVs derived from non-infected and *Mtb*-infected macrophages (40μg/mL) for 4 hrs. After PBS wash, cells were fixed with 4% PFA for 15mins at room temperature. Fixed cells were blocked with 5% normal goat serum for 1hr, and then probed with a 1/200 dilution of rabbit anti-mouse NF-κB antibody (Cell Signaling Technology) for 2hrs. FITC-conjugated goat anti-rabbit IgG at 1/200 dilution was used as the secondary Ab (Jackson ImmunoResearch) and incubated for 1hr. DAPI was used for nuclear staining at 1/200 dilution. Cover slips were mounted on slides in mounting media and observed at 40x with a Nikon c2 confocal fluorescent microscope. For data quantification, a total of approximately 100 cells in 4–5 randomly selected fields per coverslip were counted. The percentage of cells positive for nuclear NF-κB staining was calculated for each field and averaged. The Counting was performed blinded to each sample.

### RNA-Seq data analysis

Each of the six samples was first quickly checked using FastQC and then trimmed with Trimmomatic-0.30 using its included miSeq-appropriate adapter sequences, a minimum length of 36bp, and all other settings as suggested by the manual. Mapping to the mouse genome (*Mus musculus*, NCBI build 37.2) was performed using Tophat 2.0.10/bowtie2.1.0 using all forward RNAseq reads (paired and unpaired) and only paired reverse trimmed reads. Differential expression analysis was performed using cuffdiff (version 2.1.1) using the NCBI build 3.7.2 reference annotation (GTF, both biological replicates as input (4 total per comparison), and the only option using 6 threads for analysis. Significance was determined using *q* value (FDR) correction as calculated by cuffdiff for all annotated genes.

### Statistical analyses

Data were analyzed using a one-tailed paired Student t-test. Statistical significance was assumed at p<0.05.

## Results

### Exposure of endothelial cell monolayers to EVs increases monolayer permeability

**Fig 1:**
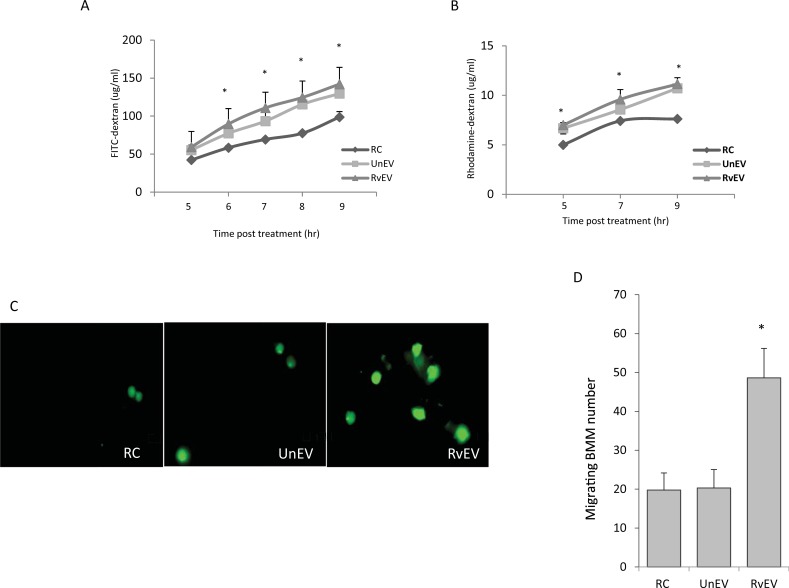
EV treatment of mouse endothelial cells causes increased cell permeability and macrophage migration. Monolayers of mouse endothelial cells (SVEC4-10) in transwell plates were treated with 40μg/mL EVs derived from non-infected or *Mtb-*infected RAW 264.7 macrophages. 4kD FITC-labeled dextran **(A)** or 70kD Rhodamine-labeled dextran **(B)** was added to top chamber and culture medium was taken from bottom chamber at various time points and the dextran concentration quantified. All the data points were generated from three independent replicates. *p<0.05 when compared to untreated endothelial cells (RC). UnEV: EVs from non-infected Raw264.7 cells, RvEV: EVs from H37Rv-infected cells. **(C)** Endothelial cell monolayers were stimulated with EVs derived from non-infected or *Mtb*-infected macrophages for 3 hours or left untreated. CFSE-labeled mouse BMMs were added to the SVEC4-10 cells. The fluorescently-labeled macrophages which migrated through the SVEC4-10 cell monolayer into the bottom of the Transwell filter were imaged 4 hrs after their addition to the SVEC-10 cells (representative images of two independent experiments). **(D)** The number of BMMs in seven randomly selected fields were counted for each condition and the total number of cells calculated. Shown is the average from two experimental replicates + SD with asterisk (*) indicating a p value < 0.05 when compared to untreated endothelial cells (RC).

To begin evaluating endothelial cell function following exposure to EVs we first assessed cell monolayer permeability upon EV treatment. The integrity of the monolayer was assessed by measuring diffusion of labeled dextran across cultured SVEC4-10 cells plated at different concentrations. A sharp drop in dextran diffusion was observed between collagen-coated transwells plated with 3.3x10^2^ and 1.65x10^3^ cells (S1 Fig). To assess cell permeability SVEC4-10 cells were plated on collagen-coated transwells at 3.3x10^3^ cells/well and treated with EVs purified from the condition media of *Mtb*-infected and uninfected Raw264.7 cells. The EV-treated SVEC4-10 cells were incubated with FITC-labeled or rhodamine-labeled dextran and the concentration of labeled dextran in the bottom chamber was measured 5 to 9 hours post-treatment. The results showed an effect of EVs on endothelial cell permeability to dextran (Fig 1A and 1B). However, there was no significant difference in permeability between treatments with EVs from non-infected and *Mtb*-infected macrophages.

### Treatment of endothelial cells with EVs from *Mtb*-infected macrophages promotes cell migration through the cell monolayer

To evaluate whether EVs released during an *Mtb* infection could facilitate cell migration through the endothelial cell monolayer, we added CFSE-labeled bone marrow-derived macrophages (BMMs) to a monolayer of SVEC4-10 cells pretreated for 3 hours with equal concentration of EVs derived from non-infected or *Mtb-*infected Raw264.7 cells. The SVEC4-10 cells showed greater than 95% viability after the different treatments and no difference in viability between EV-treated and untreated cells was observed. BMMs were also added to untreated SVEC4-10 cells as a control. Results indicate a significant increase in the number of BMMs, which had transgressed through the monolayer during the 4 hour incubation when the SVEC4-10 cells were pretreated with EVs derived from *Mtb*-infected Raw264.7 cells (Fig 1C and 1D). There was no significant difference in the number of migrating BMMs between untreated cells and those treated with EVs derived from non-infected Raw264.7 cells (Fig 1D).

### EVs derived from *Mtb*-infected macrophages upregulate gene expression and immune response-related pathways in endothelial cells

To evaluate the effects of EVs on endothelial cells more globally, we performed deep sequencing of the SVEC4-10 cellular RNA isolated 4 hours post EV treatment, and differentially expressed genes were determined. We chose 4 hours as our time point as our cell-migration data indicated that we can see a phenotypic response by the endothelial cells at 3+ hours. As shown in Table 1, at least 1.25 million paired RNA-seq reads (1.8 million overall) were obtained per sample. As expected when using the same cell type for all treatments, mapping was consistent across samples and at least ~76% of the reads were aligned and used for differential expression analysis (Table 1).

**Table 1:** Read and alignment information for each sample sequenced. Roughly 76% of all high quality reads was used for differential expression analysis.

	RC		RvEV treatment		UnEV treatment	
	Rep1	Rep 2	Rep 1	Rep 2	Rep 1	Rep 2
Paired reads	1 704 960	1 463 051	2 079 359	1 257 574	1 775 338	1 965 745
#L aligned	1 576 238	1 347 733	1 914 835	1 151 102	1 627 781	1 817 558
% total	92.5	92.1	92.1	91.5	91.7	92.5
>1 aln (%)	10	10.1	10.2	11.2	10.5	10.8
#R aligned	1 045 026	898685	1 270 323	774206	1 103 496	1 192 232
% total	61.3	61.4	61.1	61.6	62.2	60.7
> 1 aln (%)	9.1	9.2	9.4	10.2	9.7	9.8
Fwd only	773376	651079	893185	554277	739040	915923
# mapped	569385	478913	655285	403282	553004	676477
% total	73.6	73.6	73.4	72.8	74.8	73.9
>1 aln (%)	11.8	12.1	12.2	13.3	12.4	12.9
Aln Pairs	991843	850127	1 204 899	721760	1 046 516	1 133 834
% Discordant	4.2	4.5	4.4	6	4.5	4.7
% total mapped	76.3	76.2	76	75.9	76.6	76

**Fig 2:**
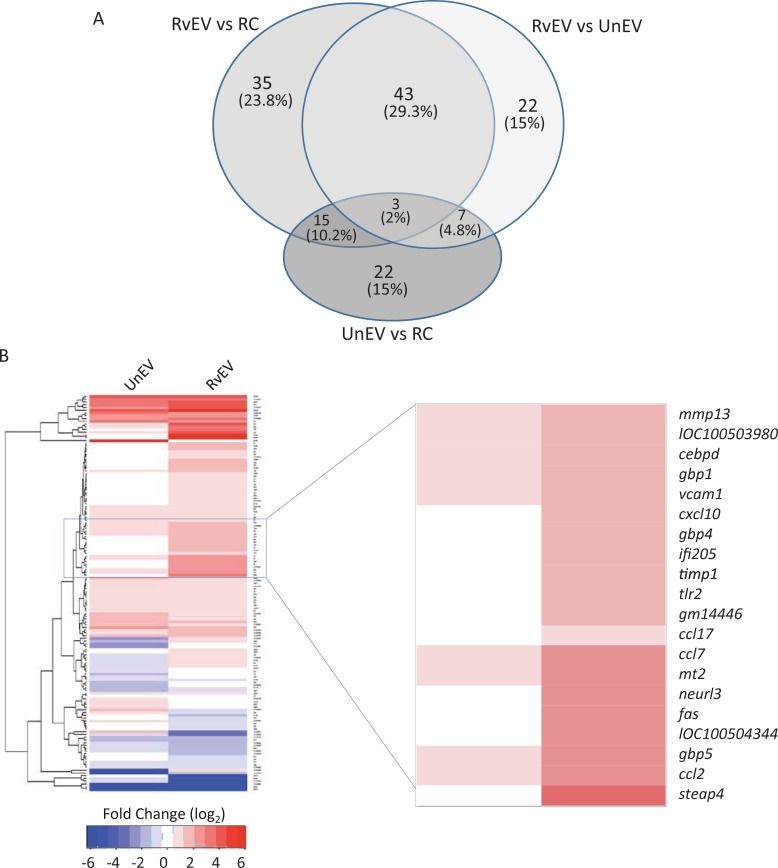
Analysis of the endothelial cell gene expression profile following treatment with EVs isolated from *Mtb*-infected and uninfected macrophages. Total RNA was sequenced for two independent biology replicates. **(A)** Venn diagram of the genes that showed a minimum two fold up- or down-regulation in endothelial cells following treatment with EVs from *Mtb*-infected (RvEV) or uninfected (UnEV) macrophages compared to each other and to untreated cells. **(B)** Hierarchical cluster analysis of differentially expressed genes in endothelial cells treated with EVs isolated from *Mtb*-infected or uninfected macrophages. The analysis was conducted with a minimal 2-fold change compared with resting endothelial cells.

Analysis of the RNA sequencing data indicated that 96 genes were expressed two fold or greater in endothelial cells treated with EVs from *Mtb*-infected macrophages relative to untreated endothelial cells, and 75 genes were expressed at least two fold higher when compared to endothelial cells treated with EVs from non-infected macrophage s (Fig 2A). The majority of the 75 genes were also found in the set of genes identified in endothelial cells treated with EVs for *Mtb*-infected compared to untreated macrophages. In contrast, we observed only 21 genes down-regulated in RvEv-treated compared to untreated cells and only 7 showed greater than a 3 fold difference (data not shown).

Hierarchical cluster analysis of these differentially expressed genes showed clusters of genes upregulated in endothelial cells treated with EVs derived from *Mtb*-infected macrophages compared to non-infected cells. One cluster included *vcam1*, *tlr2*, *cxcl10*, *ccl7* and *ccl2*, which are genes known to be involved in endothelial cell activation and immune cell migration [20, 21] (Fig 2B). To gain detailed information on the immune response-related pathways affected by EV treatment in endothelial cells, the differentially regulated genes were subjected to a pathway analysis using METACORE (https://portal.genego.com). A total of 37 pathways were found significantly upregulated when endothelial cells were treated with EVs derived from *Mtb*-infected macrophages compared to untreated cells or the cells treated with EVs from non-infected macrophages (Table 2).

**Table 2:** Shared pathways significantly upregulated in endothelial cells treated with EVs.

Rank	Pathway name	Gene found in the pathway	FDR value
1	Immune response_IL-17 signaling pathways	GRO-1, I-kB, CCL7, CCL2, NGAL	7.238894E-05
2	Immune response_Histamine H1 receptor signaling in immune response	I-kB, NFKBIA, VCAM1, MMP-13	4.287833E-04
3	Immune response_Bacterial infections in normal airways	TLR2, I-kB, FasR(CD95), MD-2	4.287833E-04
4	Immune response_HMGB1/RAGE signaling pathway	TLR2, I-kB, NFKBIA, VCAM1	4.413008E-04
5	Immune response_IL-33 signaling pathway	GRO-1, I-kB, VCAM1, CCL2	4.524081E-04
6	Immune response_TREM1 signaling pathway	TLR2, I-kB, NFKBIA, CCL2	4.524081E-04
7	Immune response_CD40 signaling	I-kB, FasR(CD95), A20, CCL2	5.253971E-04
8	Immune response_MIF-mediated glucocorticoid regulation	I-kB, NFKBIA, VCAM1	5.253971E-04
9	Immune response_Oncostatin M signaling via MAPK in mouse cells	TIMP1, MMP-13, CCL2	1.940226E-03
10	Immune response_Oncostatin M signaling via MAPK in human cells	TIMP1, MMP-13, CCL2	2.065798E-03
11	Immune response_MIF-induced cell adhesion, migration and angiogenesis	VCAM1, MMP-13, CCL2	3.611721E-03
12	Apoptosis and survival_Role of PKR in stress-induced apoptosis	I-kB, NFKBIA, FasR(CD95)	4.267708E-03
13	Immune response_HSP60 and HSP70/ TLR signaling pathway	TLR2, I-kB, MD-2	4.267708E-03
14	Immune response_TLR2 and TLR4 signaling pathways	TLR2, I-kB, MD-2	4.418892E-03
15	Immune response_Role of PKR in stress-induced antiviral cell response	TLR2, I-kB, NFKBIA	4.418892E-03
16	Immune response_IL-18 signaling	I-kB, VCAM1, CCL2	4.855339E-03
17	Chemotaxis_Leukocyte chemotaxis	I-TAC, VCAM1, IP10	8.202680E-03
18	Immune response_Oncostatin M signaling via JAK-Stat in mouse cells	TIMP1, CCL2	8.202680E-03
19	Schema: Initiation of T cell recruitment in allergic contact dermatitis	VCAM1, IP10	8.202680E-03
20	Immune response_Oncostatin M signaling via JAK-Stat in human cells	TIMP1, CCL2	9.691753E-03
21	Development_Glucocorticoid receptor signaling	NFKBIA, MMP-13	1.337917E-02
22	Development_Cross-talk between VEGF and Angiopoietin 1 signaling pathways	I-kB, VCAM1	1.504905E-02
23	Cell adhesion_Chemokines and adhesion	GRO-1, MMP-13, CCL2	1.540486E-02
24	Proteolysis_Putative SUMO-1 pathway	NFKBIA, FasR(CD95)	1.726925E-02
25	Apoptosis and survival_Caspase cascade	FasR(CD95), Caspase-4	1.923130E-02
26	CCR4-dependent immune cell chemotaxis in asthma and atopic dermatitis	VCAM1, CCL17	1.923130E-02
27	Chemotaxis_CCR4-induced chemotaxis of immune cells	VCAM1, CCL17	1.923130E-02
28	Mechanism of action of CCR4 antagonists in asthma and atopic dermatitis (Variant 1)	VCAM1, CCL17	1.923130E-02
29	Development_NOTCH1-mediated pathway for NF-KB activity modulation	I-kB, NFKBIA	1.923130E-02
30	Immune response_Lipoxins and Resolvin E1 inhibitory action on neutrophil functions	I-kB, NFKBIA	1.974668E-02
31	Immune response_HMGB1/TLR signaling pathway	TLR2, I-kB	2.026040E-02
32	Apoptosis and survival_Lymphotoxin-beta receptor signaling	I-kB, VCAM1	2.568978E-02
33	Impaired inhibitory action of lipoxins and Resolvin E1 on neutrophil functions in CF	I-kB, NFKBIA	2.568978E-02
34	Development_VEGF signaling and activation	I-kB, VCAM1	2.568978E-02
35	Immune response_IL-13 signaling via JAK-STAT	CCL2, CCL17	2.618055E-02
36	Muscle contraction_Relaxin signaling pathway	I-kB, NFKBIA	2.928011E-02
37	Transcription_NF-kB activation pathways	TLR2, I-kB	3.023603E-02

96 mouse genes (showed differential expression at least 2 fold in endothelial cells treated with EVs derived from *M*.*tb*-infected macrophages compare to resting cells) and 75 mouse genes (showed differential expression at least 2 fold in endothelial cells treated with EVs derived from *M*.*tb*-infected macrophages compare to EVs from uninfected macrophages) were selected for pathway analysis (https://portal.genego.com) respectively. 37 pathways were found significantly upregulated in both comparisons (q≤0.05). RC: Resting endothelial cells: UnEV; EVs from non-infected Raw264.7 cells; RvEV: EVs from H37Rv-infected cells.

**Fig 3:**
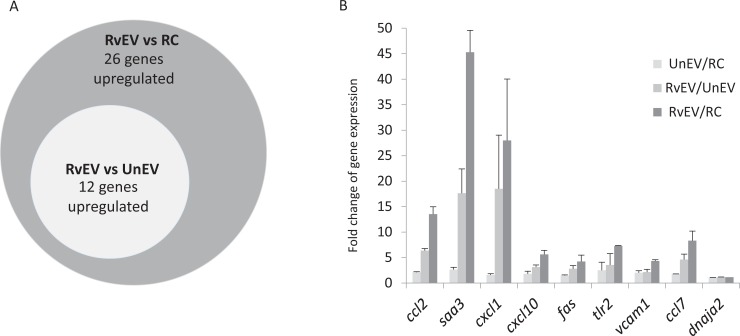
EV-induced gene expression in endothelial cells. **(A)** Endothelial cells were treated 4hrs with 40μg/mL EVs derived from either non-infected or *Mtb*-infected macrophages or left untreated. Total RNA was sequenced for two independent biology replicates. Venn diagram indicating the number of genes whose expression was >2-fold upregulated with a false discover rate (q<0.05) for each indicated comparison. **(B)** Quantitative RT-PCR was performed for a subset of genes defined as upregulated in the sequence analysis. Endothelial cells were left untreated or treated with EVs derived from uninfected or *Mtb*-infected macrophages. Total RNA was extracted followed by cDNA synthesis. Fold change of gene expression was calculated by comparative Ct method. *Dnaja2* whose expression by sequence analysis did not change before and after EV treatment was selected as a negative control. Expression data was normalized to *gapdh*. RC: Untreated cells, RvEV: Treatment with EVs from H37Rv-infected macrophages, UnEV: Treated with EVs from non-infected macrophages. Graph indicates fold change of gene expression from two independent experiments +/- SD.

A more stringent false discovery rate analysis was performed on the data resulting in the identification of 26 genes to be significantly upregulated when endothelial cells were treated with EVs from *Mtb*-infected macrophages compared to untreated cells, of which 12 were also up-regulated when compared to endothelial cells treated with EVs derived from non-infected macrophages (Fig 3A). This set also contained vascular cell adhesion molecule 1 (*vcam1*) and *tlr2*. Quantitative RT-PCR results for a subset of these 12 genes, chosen based on their known role in a host immune response, again showed a significant upregulation following exposure to EVs released from *Mtb*-infected macrophages (Fig 3B). The *dnaja2* gene expression was shown in our RNA sequence analysis to remain unchanged in SVEC4-10 cells upon EV treatment and this was confirmed by qRT-PCR (Fig 3B).

### Upregulation of VCAM1, TLR2 and CCL2 in SVEC4-10 cells upon treatment with EVs from *Mtb*-infected macrophages

**Fig 4:**
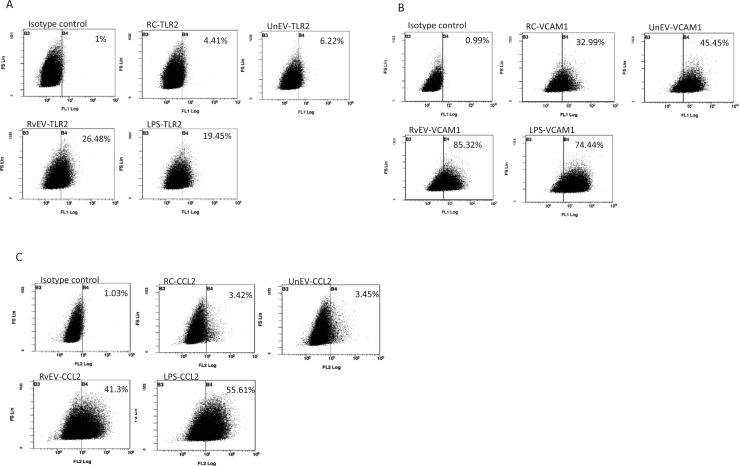
Upregulation of TLR2, VCAM-1 and CCL2 on endothelial cells following exposure to EVs from *Mtb*-infected macrophages. Endothelial cells were left untreated or treated for 16 hrs with LPS (1μg/mL) or EVs derived from non-infected or *Mtb*-infected macrophages. **(A)** Cells were stained with FITC conjugated anti-mouse TLR2 antibody or FITC conjugated anti-mouse IgG1 antibody as an isotype control. **(B)** Cells were surface-stained with FITC-labeled rat anti-mouse VCAM1 or FITC labeled anti-rat IgG2a antibody as an isotype control. **(C)** Cells were permeabilized and stained for intracellular CCL2 using PE-conjugated anti-mouse CCL2 antibody or PE-labeled IgG as an isotype control. Gates were set to approximately 1% for isotype control and were maintained for all subsequent analysis. RC: untreated cells, RvEV: Treatment with EVs from H37Rv-infected macrophages, UnEV: Treated with EVs from non-infected macrophages. Data is representative of the protein expression from three independent experiments.

Flow cytometry was performed on EV-treated SVEC4-10 cells to investigate the effects of EVs at the protein level. We evaluated the surface expression of VCAM1, TLR2 and the intracellular concentration of CCL2 as the corresponding genes showed some upregulation in SVEC4-10 cells following treatment with EVs released from *Mtb*-infected macrophages. Increased number of endothelial cells expressed surface TLR2 and VCAM1 (Fig 4A and 4B) following a 16 hour treatment with EVs derived from *Mtb*-infected macrophages compared to untreated endothelial cells or cells treated with an equal concentration of EVs from non-infected macrophages. A significant increase in the intracellular CCL2 was also observed in SVEC4-10 cells treated with EVs released from *Mtb*- infected macrophages (Fig 4C).

### Serum EVs from *Mtb*-infected mice can activate endothelial cells *ex vivo*

**Fig 5:**
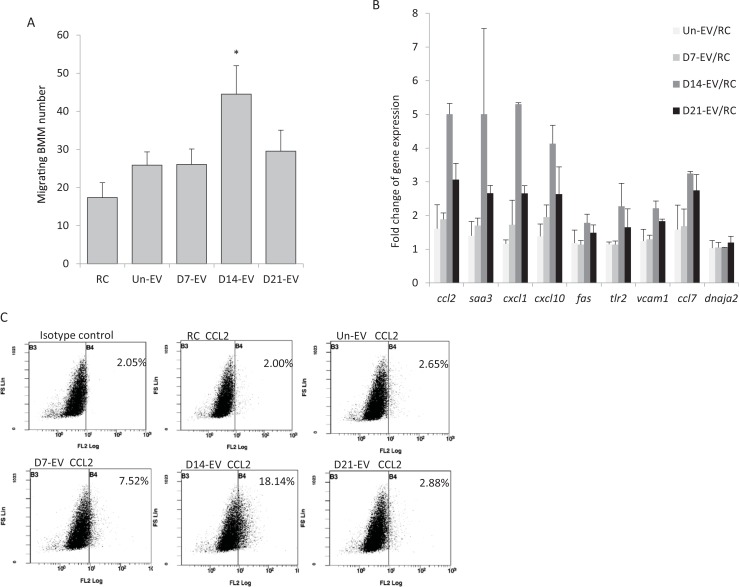
EVs derived from the serum of *Mtb*-infected mice can activate endothelial cells *ex vivo*. **(A)** SVEC4-10 cell monolayers were left untreated or stimulated for 3 hrs with EVs derived from non-infected or *Mtb*-infected mice. CFSE-labeled mouse BMMs were added to SVEC4-10 cells. The fluorescently-labeled macrophages which migrated through the SVEC4-10 cell monolayer into the bottom of the Transwell filter were imaged. The number of BMMs in seven randomly selected fields were counted and the total number of cells for each condition defined. The data is the average of three independent mouse *Mtb* infections +SD with (*) indicating a p value < 0.05 compared to RC. **(B)** Quantitative RT-PCR was performed on endothelial cells that were left untreated or treated for 4 hours with EVs derived from uninfected or *Mtb*-infected macrophages. Total RNA was extracted followed by cDNA synthesis. Fold change of gene expression was calculated by comparative Ct method. Data is from two independent mouse *Mtb* infections. **(C)** Scatter plots of flow cytometry analysis of CCL2 expression. Endothelial cells were left untreated or treated for 16 hours with EVs derived from non-infected or *Mtb*-infected macrophages. Permeabilized cells were stained with PE-conjugated anti-mouse CCL2 antibody or PE-labeled IgG as an isotype control. Gate was set for isotype control and was maintained for all subsequent analysis. RC: untreated cells. Un-EV: serum-derived EVs from uninfected mice, D7-EV, D14-EV, D21-EV: serum derived EVs from mice infected for 7, 14 and 21 days respectively. Data is representative of the CCL2 expression from two independent experiments.

Previous studies found that EV concentration in the serum increased during an *M*. *bovis* BCG and *Mtb* mouse infection [10, 11]. These results suggest that the EV pool is changing during an infection. However, the functionality of these EVs and how it varies during the course of an infection was not addressed in these studies. To evaluate serum EVs as regulators of endothelial cell function we isolated EVs from mouse serum at different time points post-infection and characterized their effect on the SVEC4-10 permeability as well as gene and protein expression. As was observed with EVs from *in vitro* infected macrophages, there was enhanced BMM migration through the endothelial cells when the SVEC4-10 monolayer was pretreated with serum-derived EVs isolated 14 days post-infection. Interestingly, we failed to see this enhanced BMM migration when we used an equal concentration of EVs isolated from serum 7 and 21 days post-infection. (Fig 5A).

To further investigate how endothelial cells respond to EVs isolated from infected mice, we characterized SVEC4-10 gene expression post EV treatment. For these studies we tested the same set of genes, 8 in total, that were found to be upregulated in endothelial cells upon treatment with EVs isolated from *Mtb* infected Raw264.7 cells. We found that a number of these genes including *ccl2*, *saa3*, *cxcl1* and *cxcl10* were upregulated in endothelial cells when treated with EVs isolated from mouse serum 14 days post *Mtb* infection when compared to untreated cells (Fig 5B). For most genes the expression levels were relatively lower when the endothelial cells were stimulated with an equal concentration of EVs isolated 7 and 21 days post-infection. As found with our *in vitro* studies, the *dnaja2* expression level did not change upon EV treatment and served as our loading control (Fig 5B). This differential expression extended to the protein level as CCL2 was expressed at the highest level in SVEC4-10 cells treated with EVs isolated 14 days post-*Mtb* infection with minimal expression above controls when using serum EVs from day 7 and 21 post-infection (Fig 5C).

### NF-κB is translocated to the nucleus in SVEC4-10 cells following treatment with EVs released from *Mtb*-infected macrophages

**Fig 6:**
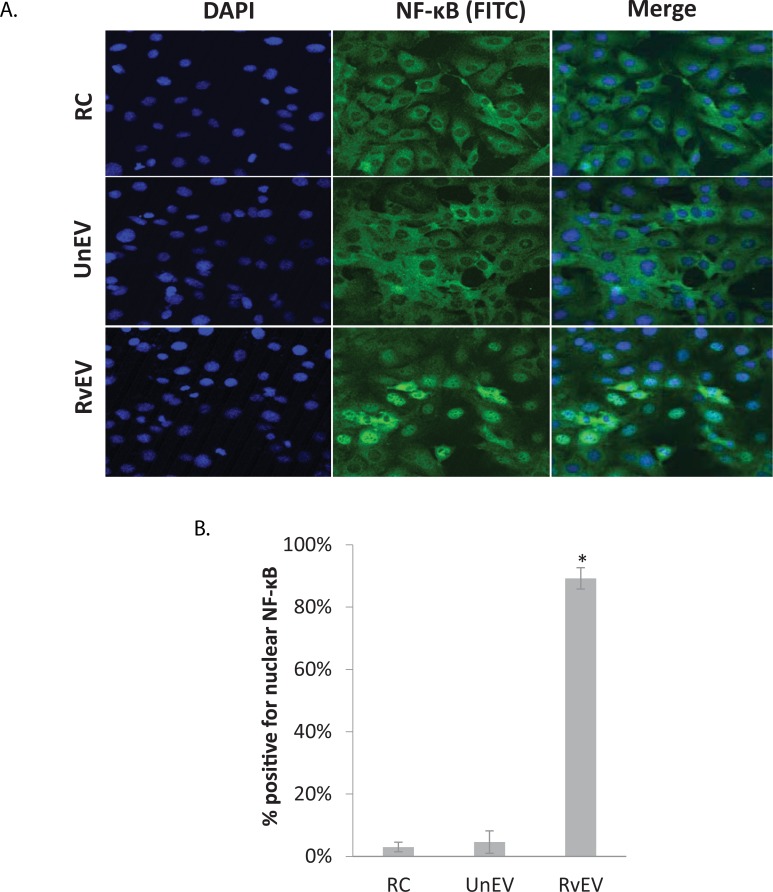
Treatment of SVEC4-10 cells with EVs from *Mtb*-infected macrophages induces NF-κB nuclear localization. SVEC4-10 cells were seeded in collagen-coated cover slips and incubated for three days to produce a cell monolayer. The cells were left untreated or treated for 4hrs with EVs released from non-infected or *Mtb*-infected macrophages (40μg/mL). Cells were fixed and stained with rabbit anti-mouse NF-κB antibody. FITC-conjugated goat anti rabbit was used as the secondary antibody. DAPI was used for nuclear staining. Cover slips were mounted on slides in mounting media and observed at 40x using a Nikon c2 confocal fluorescent microscope. **(A)** Representative images of the different treatment groups from two independent experiments. **(B)** Quantification of the number of cells with NF-κB nuclear localization. Approximately 100 cells in 4–5 randomly selected fields per coverslip were counted. (*) indicates a p value < 0.05 compared to RC or UnEV treatment. RC: untreated cells, RvEV: EVs from H37Rv-infected macrophages, UnEV: EVs from non-infected macrophages.

NF-κB is reported to control the global pro-inflammatory response in endothelial cells [22]. Therefore, to test the hypothesis that EV treatment of SVEC4-10 cells results in activation of NF-κB, we evaluated the cells for the translocation of the transcription factor to the nucleus as only activated NF-κB is present in the nucleus. After a 4hr stimulation with EVs isolated from *Mtb*-infected macrophages, we observed a significant increase in the number of endothelial cells positive for nuclear NF-κB compared to cells treated with EV derived from non-infected macrophages (Fig 6A and 6B).

## Discussion

Tuberculosis (TB) is the number one cause of death by a single infectious organism [23]. Approximately one-third of the world’s population are infected with the etiologic agent *Mycobacterium tuberculosis* with an estimated 9 million new cases and 1.5 million deaths attributed to TB annually [24]. Our inability to control the disease stem in large part from a lack of understanding of what constitutes a protective immune response. It is clear that the host must mount a TH_1_ response characterized by IFN-ɣ production and activation of naïve macrophages. However, numerous studies have shown that although this TH_1_ response is necessary for controlling an infection, it is not sufficient and there are other important players in this process such as Th_17_ cells and ɣδ T cells [25, 26]. The various cytokines such as IL-17 and IL-23 produced by these and other cells are critical for TB control [27, 28]. However, our understanding of what immune responses are necessary for controlling an *Mtb* infection is clearly incomplete [29, 30]. Recently EVs have garnered significant attention as a new mechanism of immune regulation.

EVs, which are released from most nucleated cells, are involved in intercellular communication and play an essential role in such physiological process as development, neuronal activity, cardiovascular function as well as immune regulation. EVs are also implemented in various pathological processes most thoroughly studied in the context of cancer [31] but also in diseases such as Alzheimer’s and heart disease [32, 33]. The composition and function of the different EVs varies and depends on the cell of origin and the physiological state of the cell at the time of EV release.

Our understanding of how EVs regulate the immune response during an infection is limited but studies have shown that EVs can carry pathogen-derived molecules including proteins, lipids and even pathogen nucleic acids [2, 34–35]. Therefore, since EVs are present in circulating bodily fluids including blood they have the potential to carry these microbial components to sites distal to the infection, which can result in a more systemic effect. One of the most exposed tissues to EVs in circulation is the endothelium.

The endothelium which is composed of 1 to 6 × 10^13^ endothelial cells forms the inner lining of the blood vessels and the lymphatic system. Due to its unique location and the large surface area that is 4,000 to 7,000 m^2^, the endothelium is in constant contact with circulating blood [36]. Thus, endothelial cells are among the first cells exposed to pathogens or pathogen-derived stimuli, including circulating EVs. Previous work from our laboratory and others determined that during an infection, the concentration of circulating vesicles increases [10–11, 37]. Understanding the interaction between EVs and endothelial cells during an *Mtb* infection will contribute to our knowledge of how the host responds to infection. In the current study, we observed a significant upregulation of endothelial cell genes involved in adhesion and inflammation when endothelial cells were exposed to EVs derived from *Mtb*-infected macrophages and several immune response-related pathways were up-regulated. We also found from our *ex-vivo* studies that EVs released from mice 14 days post-infection were capable of inducing endothelial cell activation similarly to the EVs isolated from *in vitro* infected macrophages. Taken together, the present study indicates that EVs can activate endothelial cells and that the interaction between EVs and endothelial cells can impact the host immune response during an *Mtb* infection.

Intercellular junctions between endothelial cells mediate the barrier function between the vascular lumen and the vascular wall. The endothelium is reported to become leaky in pathogenic conditions due to destabilized junctions and increased junctional permeability [38–40]. Our permeability assay with fluorescent dyes indicates that, when compared to an untreated endothelial cell monolayer, EV treatment significantly disrupted integrity of the cellular tight junctions. This was observed at EV concentrations of 40μg/ml but not 10 μg/ml (data not shown). However, we failed to observe a difference in EVs isolated from infected and uninfected macrophages. This observation may be due to a diminished permeability responsiveness by the endothelial cells. Bonner et al [41] reported that permeability responsiveness of endothelial cells from human umbilical cord veins (HUVEC) to dengue viral infection was maximal at 2 and 3 days after seeding and declined over a period of 7 days. In our study, to ensure the formation of intact monolayer, we cultured ECs for 6 days. Potentially, shorter culture duration and methods with higher sensitivity such as electrical resistance measurements could potentially define differences between the two EV populations.

During an infection the endothelium interacts with various pathogens including viruses and bacteria, as well as to inflammatory leukocytes and the mediators they release [12, 42–43]. Activated ECs, in turn, impact the outcome of the inflammatory response by regulating leukocytes migration through releasing a number of cytokines/chemokines and expression of cellular adhesion molecules (CAMs). Therefore, it is important to understand how the endothelium is affected during an *Mtb* infection. Feng et al [44] has shown that during an *Mtb* infection, VCAM1 expression is upregulated and that the expression is maintained in the lung over the course of the infection, supporting the idea that VCAM-1 actively facilitates inflammatory T cells migration to the infection site. Our studies indicate that EVs produced during an *Mtb* infection are capable of activating endothelial cells, upregulating immune-response related genes, including *ccl2*, *vcam1*, and *cxcl1* as well as pathways, such as cytokine-cytokine receptor interaction, B cell receptor signaling pathway and toll-like receptor signaling pathway (Table 2). This activation extends to the protein level as we observed chemokines and adhesion molecules, such as CCL2 and VCAM1 to be upregulated, likely promoting the macrophage trans-endothelial migration we observed.

The composition of EV in circulation is dynamic; which cells are releasing EVs, what type of EVs are being released (microvesicles, exosomes or apoptotic bodies) and changes in the molecular composition of the EV from a given cell type [45] change over time. We know little about these changes in EV composition during the various stages of an *Mtb* infection. Recently, a panel of *Mtb* peptides were identified in EVs derived from sera of TB patients and LTBI individuals, indicating that *Mtb* infection can modify EV composition (5). *Mtb* infection can also lead to significant changes in the protein composition of EVs derived from *Mtb*-infected THP-1-derived macrophages [46]. Interestingly our *in vivo* experiments indicate that EVs isolate from mouse serum 14 days post-infection were more stimulatory in promoting endothelial cell activation compared to EVs isolated 7 and 21 days post infection. We also observed that EVs isolated from mouse serum at days 10 and 15 were more active in promoting an antigen specific T cell response than EVs isolated 21 days post infection [10]. These results suggest that EVs may be the most immunological active during the initial stages of an *Mtb* mouse infection.

We have previously shown that *Mtb* proteins are present in EVs derived from *Mtb*-infected macrophages and both *in vitro* and *in vivo*-derived EVs promoted increased TNF-α production in bone-marrow derived macrophages [10–11, 47]. The expression of TNF-α was dependent on the activation of NF-κB, which is a transcription factor linked to the pro-inflammatory response in endothelial cells [22]. The cross-regulation between the Type 1 interferon and NF-κB pathways was also found during viral infections [48] and later supported by in silico study that identified binding sites for both factors in the promoters of proinflammatory genes [49]. We observed significant higher number of nuclear translocation events for both NF-κB but not IRF3 (data did not show) in endothelial cells treated with EVs derived from *Mtb*-infected macrophages. The result suggests the involvement of NF-κB pathways in endothelial cell activation by EVs derived from *Mtb*-infected macrophages. However, at present we don’t know the composition of these EV at these different time points and what components, host or bacterial, are driving endothelial cell activation. Potential candidates include *Mtb* protein components such as the Antigen 85 complex or lipoproteins such as the LpqH, which previous studies have shown to be present on EVs released from *Mtb* infected macrophages and can activate macrophages through TLR2 [8].

In summary, endothelial cells are exposed to EVs during an *Mtb* infection and this exposure results in upregulation of genes and proteins known to promote recruitment and activation of leukocytes. The importance of this endothelial cell activation in controlling an *Mtb* infection or the inflammatory response awaits further study. However, considering the importance of endothelial cells in leukocyte function, the effect is likely significant. Future studies are needed to define the cell populations which are producing the EVs and how this changes over the course of an infection as well as define the components on EVs that are functioning to activate the endothelial cells.

## Supporting information

S1 Fig Endothelial cell monolayer formation assay. Different cell concentrations were plated in a transwell plate and the cell numbers necessary to form an intact monolayer that can block over 90% of dye diffusion through the transwell were defined.
(PDF)

S1 Table Primers for quantitative PCR assay. The table contains the primer sequences for all the endothelial cell genes evaluated in this study.
(PDF)
