# Supplementary material for: Activation of endothelial cells by extracellular vesicles derived from Mycobacterium tuberculosis infected macrophages or mice
Source: PLoS One. 2018 May 31;13(5):e0198337. doi: 10.1371/journal.pone.0198337 (PMC5979010; doi:10.1371/journal.pone.0198337)
Supplement: S1 Fig — Different cell concentrations were plated in a transwell plate and the cell numbers necessary to form an intact monolayer that can block over 90% of dye diffusion through the transwell were defined. (PDF) [file pone.0198337.s001.pdf]

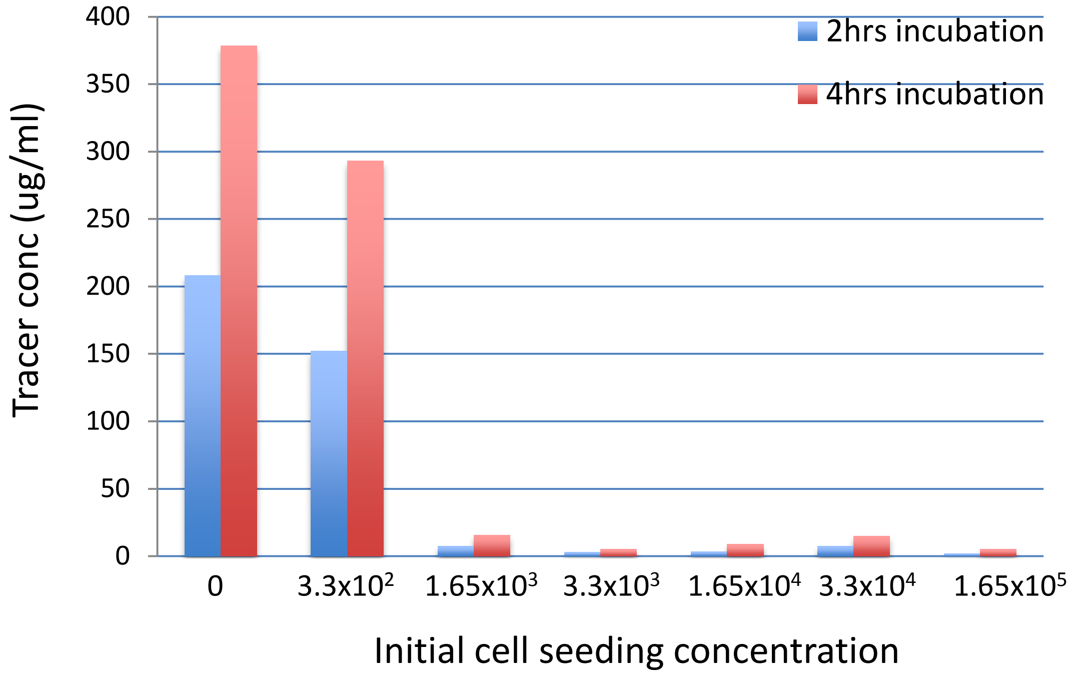

S1 Figure: Evaluating endothelial cell monolayer formation at different cell concentrations. Mouse endothelial cells (SVEC4-10) were seeded at various conentrations into top chamber of a transwell and cultured for 6 days. Rhodamine-B dextran (70kD, 1mg/mL) was added to the top chamber and incubated for 2 and 4hrs. Media was taken from bottom chamber to determine dye concentration.
