# Supplementary material for: Activation of endothelial cells by extracellular vesicles derived from Mycobacterium tuberculosis infected macrophages or mice
Source: PLoS One. 2018 May 31;13(5):e0198337. doi: 10.1371/journal.pone.0198337 (PMC5979010; doi:10.1371/journal.pone.0198337)
Supplement: S1 Table — The table contains the primer sequences for all the endothelial cell genes evaluated in this study. (PDF) [file pone.0198337.s002.pdf]

S1 table: Primer sequences for Real-time PCR

Gene	Primer sequence	
ccl2	Forward	5' CCTGCTGTTCACAGTTGCC 3'
	Reverse	5' ATTGGGATCATCTTGCTGGT 3'
ccl7	Forward	5' TCTGTGCCTGCTGCTCATAG 3'
	Reverse	5' TTCCTCTTGGGGATCTTTTG 3'
cxcl1	Forward	5' CCACACTCAAGAATGGTCGC 3'
	Reverse	5' TCTCCGTTACTTGGGGACAC 3'
cxcl10	Forward	5' CTCATCCTGCTGGGTCTGAG 3'
	Reverse	5' CCTATGGCCCTCATTCTCAC 3'
fas	Forward	5' CAGACATGCTGTGGATCTGG 3'
	Reverse	5' CCTCAGCTTTAAACTCTCGGA 3'
saa3	Forward	5' TCCATTGCCATCATTCTTTG 3'
	Reverse	5' AGTAGGCTCGCCACATGTCT 3'
tlr2	Forward	5' CATCACCGGTCAGAAAACAA 3'
	Reverse	5' ACCAAGATCCAGAAGAGCCA 3'
vcam1	Forward	5' CCGGCATATACGAGTGTGAA 3'
	Reverse	5' TCGGGCGAAAAATAGTCCTT 3'
dnaja2	Forward	5' AGGATTACGTTCACTGGGGA 3'
	Reverse	5' TCCCATCTCTCTGGAACACC 3'
gapdh	Forward	5' TCGTCCCGTAGACAAAATGG 3'
	Reverse	5' TTGAGGTCAATGAAGGGGTC 3'
